# The Sweet Drive Test: refining phenotypic characterization of anhedonic behavior in rodents

**DOI:** 10.3389/fnbeh.2014.00074

**Published:** 2014-03-07

**Authors:** António Mateus-Pinheiro, Patrícia Patrício, Nuno D. Alves, Ana R. Machado-Santos, Monica Morais, João M. Bessa, Nuno Sousa, Luisa Pinto

**Affiliations:** ^1^School of Health Sciences, Life and Health Sciences Research Institute (ICVS), University of MinhoBraga, Portugal; ^2^ICVS/3B's - PT Government Associate LaboratoryBraga/Guimarães, Portugal

**Keywords:** depression, anhedonia, Sweet Drive Test, ultrasonic vocalizations, antidepressants, sucrose consumption test

## Abstract

Measuring anhedonic behavior in rodents is a challenging task as current methods display only moderate sensitivity to detect anhedonic phenotype and, consequently, results from different labs are frequently incongruent. Herein we present a newly-developed test, the Sweet Drive Test (SDT), which integrates food preference measurement in a non-aversive environment, with ultrasonic vocalizations (USVs) recording. Animals were placed in a soundproofed black arena, under red light illumination, and allowed to choose between regular and sweet food pellets. During the test trials, 50 KHz USVs, previously described to be associated with positive experiences, were recorded. In a first experimental approach, we demonstrate the ability of SDT to accurately characterize anhedonic behavior in animals chronically exposed to stress. In a subsequent set of experiments, we show that this paradigm has high sensitivity to detect mood-improving effects of antidepressants. The combined analysis of both food preference and the number of 50 KHz vocalizations in the SDT provides also a valuable tool to discriminate animals that responded to treatment from non-responder animals.

## Introduction

Animal models of depression are valuable tools to better elucidate the neuropathological basis of depressive spectrum disorders, and to provide insights into antidepressants mechanisms of action, as well as into the identification of new putative therapeutic targets (Cryan et al., 2002; Berton et al., 2012). However, it is important to consider that the translational potential of rodent models of depression relies not only on the capacity to reproduce the etiology and core pathological marks of the disease in the animals, but also in the ability to properly measure and characterize key behavioral hallmarks of depression. Anhedonia (i.e., a relative lack of pleasure in response to a formerly rewarding stimuli) is a cardinal hallmark of several forms of depression (typical and atypical major depression, dysthymic disorder or melancholic depression) (American Psychiatric Association, 2013) and it is therefore highly relevant to accurately characterize anhedonic behavior in rodent models of psychiatric disorders, such as depression. The gold-standard methods to characterize hedonic behavior involve the preference for highly palatable substances, in the usually called “sucrose (or saccharine) consumption test” (SCT); in these tests, preference for a sweetened solution, in relation to water, is assessed (Papp et al., 1991; Tye et al., 2013). However, several authors raised concerns regarding the interpretation of reduced sucrose consumption as a manifestation of anhedonia, due to confounding factors such as the long-lasting simultaneous food- and water-deprivation periods preceding the test (usually from 18 to 24 h), its moderate sensitivity do discriminate anhedonic behavior, its variability between different strains and due to the unreliability of the procedure among laboratories (Konkle et al., 2003; Anisman and Matheson, 2005; Der-Avakian and Markou, 2012; Stuart et al., 2013). Different labs have put forward alternative methods to measure anhedonic behavior in rodents (Surget et al., 2011; Stuart et al., 2013), ranging from the characterization of behavioral traits that have been arguably correlatable with the manifestation of anhedonia for instance, submissive behavior (Strekalova et al., 2004) or reduced sexual activity (Gronli et al., 2005) to more elaborate protocols involving intracranial self stimulation (ICSS) in goal-directed test paradigms (Harrison et al., 2001; Stoker and Markou, 2011).

In the attempt to refine and complement current approaches, we developed a test to characterize anhedonic behavior in rats based on the simultaneous assessment of food preference and ultrasonic vocalizations (USVs) recording- the Sweet Drive Test (SDT). Previous studies have shown that rodents USVs patterns provide important information regarding context perception and the emotional status of the animals. In particular, while USVs in the 20–22 KHz frequencies band have been associated to negative and aversive experiences, such as anxiety, fear or even pain perception, 50 KHz vocalizations have been associated to positive and pleasurable experiences. In light of these observations, we incorporated ultrasound microphones in the SDT apparatus to record USVs during test trials and to assess whether it could reinforce the assessment of hedonic behavior in rodents.

In a first set of experiments, we sought to validate SDT in an animal model of depression based in the exposure to unpredictable chronic mild stress (uCMS). In a subsequent experimental set, we assessed SDT sensitivity to characterize the mood-improving effects of two antidepressants (fluoxetine and imipramine) and compared it to the gold-standard method, SCT.

## Materials and methods

### Animals and treatments

Two month-old male Wistar rats, weighing 200–250 g (Charles-River Laboratories) were maintained under standard laboratory conditions (12 h light: 12 h dark cycles with the dark phase beginning at 8 pm, 22°C, relative humidity of 55%, *ad libitum* access to food and water). Groups of rats (*n* = 10–18 per group) were randomly assigned to the following four experimental groups: non-stress control + saline; stress (uCMS) + saline; uCMS + fluoxetine and uCMS + imipramine. Additionally, 2 month-old Sprague Dawley male and female rats (Control + saline and uCMS + saline) were also used to assess the reproducibility and usefulness of the test in other strains and in female rodents. In the first set of experiments, an uCMS protocol was applied for 4 weeks, accordingly to what was previously validated and described (Bessa et al., 2009). In the second experimental set, animals were exposed to uCMS during 6 weeks and antidepressants (ADs) fluoxetine (10 mg.kg^−1^; Kemprotec) and imipramine (10 mg.kg^−1^; Sigma-Aldrich) were administered intraperitoneally (1 ml.kg^−1^) everyday, during the 2 last weeks of the uCMS protocol. Weight gain and food intake were measured weekly during the entire protocol to monitor eventual changes induced by uCMS. All behavioral tests were conducted during the animals' nocturnal activity period. All procedures were carried out in accordance with EU Directive 2010/63/EU and NIH guidelines on animal care and experimentation.

### Sweet drive test (SDT)

#### SDT arena

**Figure 1:**
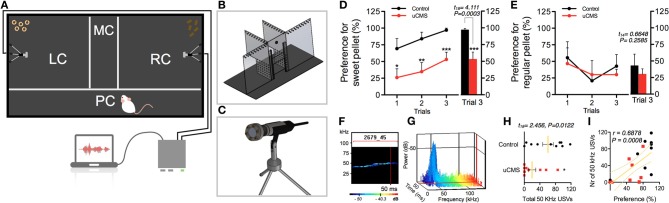
**Characterization of anhedonic behavior in rats exposed to uCMS, in the Sweet-Drive Test (SDT) paradigm. (A)** Preference for sweet pellets was measured in a compartmentalized SDT apparatus **(B)**, in which ultrasonic vocalizations were recorded using ultrasound microphones **(C)**. **(D,E)** Preference values, along 3 trials, in a sweet pellet vs. regular pellet paradigm **(D)** and in a regular pellet vs. regular pellet paradigm **(E)**. **(F–I)** The total number of 50 KHz vocalizations was measured **(F,H)** and correlated with preference values **(I)**. ^*^P < 0.05, ^**^P < 0.01, ^***^P < 0.001. Error bars, s.e.m., *n* = 8–10.

The SDT apparatus consists in a black acrylic enclosed arena (L 82 cm × W 44 cm × H 30 cm), divided by transparent and perforated walls that defined 4 separated chambers (Figures 1A,B and Movie 1): a pre-chamber (PC; L 82 cm × W 12 cm × H 30 cm) in which the animal is initially placed, which is connected by an automatic trap-door to a middle chamber (MC; L 20 cm × W 30 cm × H 30 cm); once the animal enters the MC, the trap-door closes, and the animal is allowed to move freely between the right- (RC) and left-chambers (LC) (86 × 50 × 30 cm). The arena has a transparent acrylic lid used in every trial to provide noise-reduction.

#### Ultrasonic vocalizations (USVs)

The RC and LC are equipped with ultrasound microphones (Figure 1C), so that animals' USVs can be recorded during trials. Ultrasound Microphones (CM16/CMPA, Avisoft Bioacoustics) sensitive to frequencies of 10–200 KHz were used, 20 cm above the floor, in all experiences. These were connected via an Avisoft UltrasoundGate 416H (Avisoft Biocoustics) to a personal computer. Vocalizations were recorded using the Avisoft-Recorder (version 5.1.04) with the following settings: sampling rate: 250,000; format: 16 bit. All 50 KHz vocalizations, identified by automatic data processing, were individually analyzed and validated by the experimenters. The total number of 50 KHz vocalizations emitted was assessed.

#### Experimental protocol

Animals were pre-habituated to sweet pellets (Cheerios®, Nestlé) in two different days, 4 and 2 weeks before the test, during the animals' activity period (60 Cheerios per cage evenly distributed among the corners of the cage); during periods of habituation to the sweet pellets, exposure to mild stressors was suspended. Furthermore, animals were firstly habituated to the SDT apparatus in the day preceding the first trial, for 6 min. In each testing day, food was removed specifically during the animals' inactive period, only 10 h before the test trial (to avoid test execution in an eventually confounding starvation state, but also to preclude odor and/or taste pre-conditioning to the usual regular food). Three SDT trials were conducted (1 trial every 48 h). Trials started at 8:30 pm (30 min after the beginning of the animals' nocturnal activity period) and were performed under red light. Before initiating a new trial, the SDT arena was carefully cleaned with ethanol 10%. Twenty pre-weighted regular food pellets (Mucedola 4RF21-GLP; 53.3% Carbohydrates, 3700 Kcal.Kg^−1^) were placed in the LC, and 20 pre-weighted Cheerios® (68% Carbohydrates, 3800 Kcal.Kg^−1^) pellets were placed in the RC. After placing the animals in the PC, the transparent acrylic lid was placed. At this moment video and USVs recording were started. Once the animal crossed the trap-door and entered the MC, this door closed and the animal was allowed to freely explore the LC and the RC, for 10 min. At the end of the trial, pellets consumption was determined and preference for sweet pellets (Cheerios® pellets) was determined as follows: preference for sweet pellets (%) = Consumption of Sweet Pellets (g)/Total Food Consumption (g) × 100.

Video recording allowed to assess exploratory parameters, namely the “first choice” (first chamber in which the animal entered), “time in the pre-chamber” (latency to enter to the MC), “number of incursions” (number of entrances in each chamber) and evaluate whether these measures could differentially affect the test performance in different experimental groups. USVs analysis allowed to assess the total number of “positive” 50 KHz vocalizations during trials, and to correlate these values with the evaluated preference values.

### Sucrose consumption test (SCT)

Anhedonia was assessed on weeks 4 and 6 of the uCMS protocol using the gold standard sucrose consumption test. Baseline sucrose preference values were established during a 1-week habituation period. The test consists in the presentation of two pre-weighed drinking bottles, containing water or 2% (m/v) sucrose solution for 1 h. Before each test, rats were food and water-deprived for 12 h. Sucrose preference was calculated according to the formula: sucrose preference = (sucrose intake)/(sucrose intake + water intake) × 100, as previously described (Bessa et al., 2009). SCT was performed during the nocturnal activity period of animals (starting at 8.30 p.m), 24 h after the third trial of SDT; after the SDT trial, animals were allowed to feed freely until 12 h preceding SCT.

### Novelty suppressed feeding test (NSF)

Anxious-like behavior was assessed through the NSF test at the end of the uCMS protocol. After an 18 h food-deprivation period, animals were placed in an open-field arena, where a single food pellet was positioned in the center, as previously described (Bessa et al., 2009). After reaching the pellet, animals were individually returned to their home cage, where pre-weighed food was available, and were allowed to feed for 10 min. The latency to feed in the open-field arena was used as an anxiety-like behavior index, whereas the food consumption in the home cage provided a measure of appetite drive.

### Open-field test (OF)

The open field (OF) test was used as an additional measure of anxious-like behavior, as well as to evaluate locomotor performance and exploratory activity. The open field apparatus consisted of a brightly illuminated square arena of 43.2 × 43.2 cm closed by a wall of 30.5 cm high. Rats were placed individually in the center of the open field arena and their movement was traced, for 5 min, using a two 16-beam infrared system. The resulting data was analyzed using the Activity Monitor software (Med Associates, Inc.), considering two previously defined areas: a central and an outer area. Distance traveled in each of the zones was recorded and analyzed. The ratio between the distance traveled in the center and in the periphery of arena was used as indicative of anxiety-like traits.

### Novel object recognition test (NOR)

Cognitive function was assessed in the NOR test. Briefly, rats were first habituated to the testing arena for 10 min. On the next day, each animal was allowed to explore two identical objects placed in the arena for 10 min. One hour later, rats explored the same arena for 5 min, with one of the familiar objects replaced by a novel object. Recognition memory was expressed by the discrimination index (D), which was defined as D = (time of exploration novel object − time of exploration familiar object)/total time of exploration.

### Forced swimming test (FST)

Learned-helplessness was assessed using the forced swimming test. Assays were conducted 24 h after a 5-min pretest session, by placing the rats in transparent cylinders filled with water (25°C; 50 cm depth) for 5 min. Trials were video-recorded and the immobility time as well as the latency to immobility were measured using an automated video tracking system (Viewpoint). Learned-helplessness was considered as an increase in the immobility time and a decrease in the latency to immobility.

### BrdU immunostaining

In the last day of behavioral testing, animals were given single intraperitoneal injection of Bromo-deoxyuridine (BrdU, Sigma-Aldrich, 100 mg.kg^−1^; thymidine analog that incorporates into DNA during the S-phase of the mitotic process). Twenty-four hours after the injection, animals were deeply anaesthetized with sodium pentobarbital (20%; Eutasil, Sanofi) and were transcardially perfused with cold 4% paraformaldehyde (PFA). Brains were removed and post-fixed in 4% PFA. Serial coronal cryosections (20 μm) were cut and stained for BrdU (1:50; Dako). Secondary antibody Alexa Fluor® 488 (Molecular Probes) was used for detection. Nuclei were counterstained using DAPI. Proliferation densities were estimated in the subgranular zone (SGZ; defined as a two-cell layer-thick zone on the inner side of the granule cell layer of the dentate gyrus), using a confocal microscope (Olympus FV1000).

### Morphological analysis

For the three-dimensional morphometric analysis, four animals from each group were deeply anaesthetized with sodium pentobarbital (20%; Eutasil, Sanofi), transcardially perfused with 0.9% saline and processed. Briefly, brains were immersed in Golgi-Cox solution for 21 days; transferred to a 30% sucrose solution and cut on a vibratome. Coronal sections (200 μm) were collected in 6% sucrose and blotted dry onto gelatin-coated microscope slides. They were subsequently alkalinized in 18.7% ammonia, developed in Dektol (Kodak), fixed in Kodak Rapid Fix, dehydrated and xylene cleared before coverslipping. Dendritic arborization was analyzed in the dentate gyrus. For each selected neuron, all branches of the dendritic tree were reconstructed at 1000x (oil) magnification using a motorized microscope (BX51, Olympus) and Neurolucida software (Microbrightfield). A three-dimensional analysis of the reconstructed neurons was performed using NeuroExplorer software (Microbrightfield). For each animal, 10 neurons were studied and total dendritic length was determined.

### Corticosterone levels measurements

Corticosterone levels were measured in blood serum, collected by tail venopuncture, using a commercially available ELISA kit (R&D Systems). Sampling was performed between 8 and 9 am (nadir) and between 8 and 9 pm (zenith; peak) at the end of the uCMS protocol. Samples were run in duplicate.

### RT-PCR measurements

mRNA expression levels of the genes involved in the mediation of anhedonia were determined by qRT-PCR in the nucleus accumbens (NAc) and pre-frontal cortex (PFC) from six animals of each group (CT and CMS + Sal). Total RNA (500 ng) was reverse transcribed using qScript™ cDNA SuperMix (Quanta Biosciences™). Oligonucleotide primers for dopamine receptor 1 (Drd1), 2 (Drd2) and 3 (Drd3), prodynorphin (Pdyn), opioid receptor μ1 (Oprm1) and cannabinoid receptor 1 (Cnr1) were designed using Primer-BLAST software (NCBI). Sense and antisense sequences can be found in Table 1. Real time reactions were performed in an Applied Biosystems 7500 Fast Real-Time PCR System (Applied Biosystems) using 5x HOT FIREPol® EvaGreen® qPCR Mix Plus (ROX) (Solis Biodyne). The housekeeping gene Beta-2-Microglobulin (B2M) was used as internal control for normalization of the target gene's expression. The relative expression was calculated using the Δ Δ Ct method. Results are presented as relative expression of the target gene.

**Table 1:** **Sense and antisense sequences of oligonucleotide primers used in the qRT-PCR**.

**Gene**	**Sense**	**Antisense**	**Product size (bp)**
*B2M*	GTGCTTGCCATTCAGAAAACTCC	AGGTGGGTGGAACTGAGACA	136
*Drd1*	TCCTTCAAGAGGGAGACGAA	CCACAAACACATCGAAGG	168
*Drd2*	ATGTGCTGGTGTGCATGGCT	CACCCACCACCTCCAGGTAGAC	142
*Drd3*	GGGGTGACTGTCCTGGTCTA	TGGCCCTTATTGAAAACTGC	169
*Pdyn*	CCTGTCCTTGTGTTCCCTGT	AGAGGCAGTCAGGGTGAGAA	157
*Oprm1*	CAACTTGTCCCACGTTGATG	TAATGGCTGTGACCATGGAA	119
*Cnr1*	AGGAGCAAGGACCTGAGACA	TAACGGTGCTCTTGATGCAG	166

### Statistical analysis

Statistical analyses were done using SPSS software (SPSS, Chicago, IL, USA). *t*-test was used to evaluate differences between two groups where appropriate. ADs effects on different behavioral dimensions were evaluated using One-Way analysis of variance (ANOVA). F-values and P-values derived from the between groups analysis of variance are properly indicated in the figures. Differences between groups were determined by Bonferroni's *post-hoc* multiple comparison test, and the corresponding P-values are depicted in the figures. Statistical significance was accepted for *P* < 0.05.

## Results

To assess the efficacy of the new behavioral paradigm described herein, we used an uCMS exposure model (Bessa et al., 2009) in Wistar-Han male rats. This protocol is known to induce core symptoms of depression in rodents, including anhedonia (Bessa et al., 2009). In a first approach, we aimed to evaluate whether SDT could discriminate the impacts of uCMS exposure in hedonic behavior of animals chronically exposed to stress during 4 weeks (Figure 1). In the tested paradigm, control (non-stressed) animals developed high preference for sweet pellets over the three trials, reaching over 95% of preference in trial 3; conversely, uCMS-exposed animals evidenced no significant preference for sweet food pellets over regular food pellets (preference values ≈50%) (Figure 1D). This group discrimination based on SDT performance was absent when using a neutral regular food vs. regular food test paradigm (Figure 1E).

In order to have a multi-parametric measurement of anhedonic behavior we complement the food preference analysis with simultaneous recording of 50 KHz USVs (Figures 1F,G). Remarkably, results showed that animals with reduced preference for sweet pellets, also presented a reduction in the number of 50 KHz “positive” vocalizations during the test (Figure 1H). In fact, the frequency of vocalizations correlated positively with the preference for sweet pellets (Figure 1I), and allowed to effectively discriminate between control and uCMS animals.

**Figure 2:**
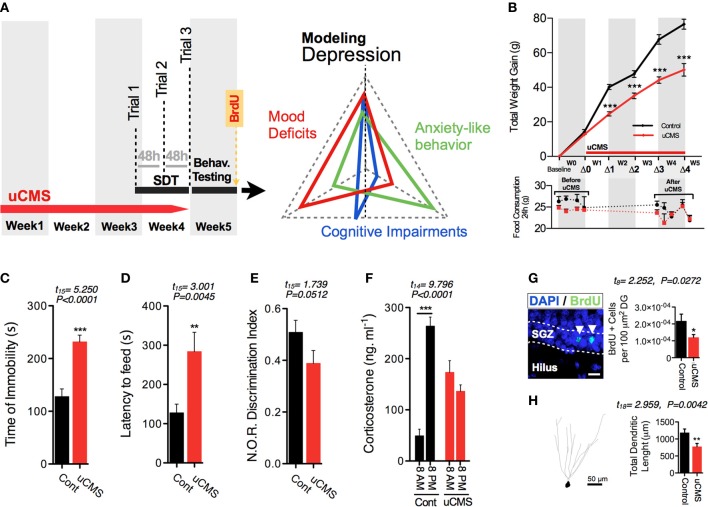
**Effects of uCMS exposure in different physiological and behavioral dimensions. (A)** After 4 weeks of uCMS exposure, multi-dimensional depressive-like behavior was assessed. **(B)** Weight gain and food intake, before and after 4 weeks of uCMS. Animals exposed to uCMS presented a decreased total body weight gain during stress exposure [Week1 (Δ1): *t*_(15)_ = 7.969, *P* < 0.0001; Week2 (Δ2): *t*_(15)_ = 4.948, *P* < 0.0001; Week3 (Δ3): *t*_(15)_ = 6.877, *P* < 0.0001; Week4 (Δ4): *t*_(15)_ = 5.782, *P* < 0.0001]. Daily food consumption of uCMS animals remained unaltered after stress exposure. Animals were tested in the Forced Swimming Test **(C)**, Novelty Suppressed Feeding **(D)** and Novel Object Recognition **(E)** test paradigms. ^*^P < 0.05, ^**^P < 0.01, ^***^P < 0.001. Error bars, s.e.m., *n* = 8–10. **(F–H)** Corticosterone serum levels **(F)**, hippocampal proliferation **(G)** and dendritic morphology of hippocampal granular neurons **(H)** were assessed. ^*^P < 0.05, ^**^P < 0.01, ^***^P < 0.001. Error bars, s.e.m., *n* = 5–10.

Evaluation of anhedonia with SDT was subsequently complemented with additional phenotypic characterization of animals that revealed multi-dimensional physiological and behavioral deficits normally encompassed in the symptomatic profile of depressive disorders (Bessa et al., 2009; Mateus-Pinheiro et al., 2013) (Figure 2A). As previously reported, uCMS exposure induced a significant reduction in total body weight gain, but no significant alterations were found in the total daily food consumption between control and uCMS-exposed animals either before, or after 4 weeks of stress exposure (Figure 2B). Moreover, uCMS exposure promoted the emergence of behavioral despair signs (Figure 2C), heightened anxiety-like behavior (Figure 2D) and cognitive disabilities (Figure 2E). These behavioral changes were accompanied with the disruption of normal corticosterone serum levels (Figure 2F), a decrease in overall hippocampal cell proliferation (Figure 2G) and dendritic atrophy of dorsal hippocampal granular neurons (Figure 2H).

**Figure 3:**
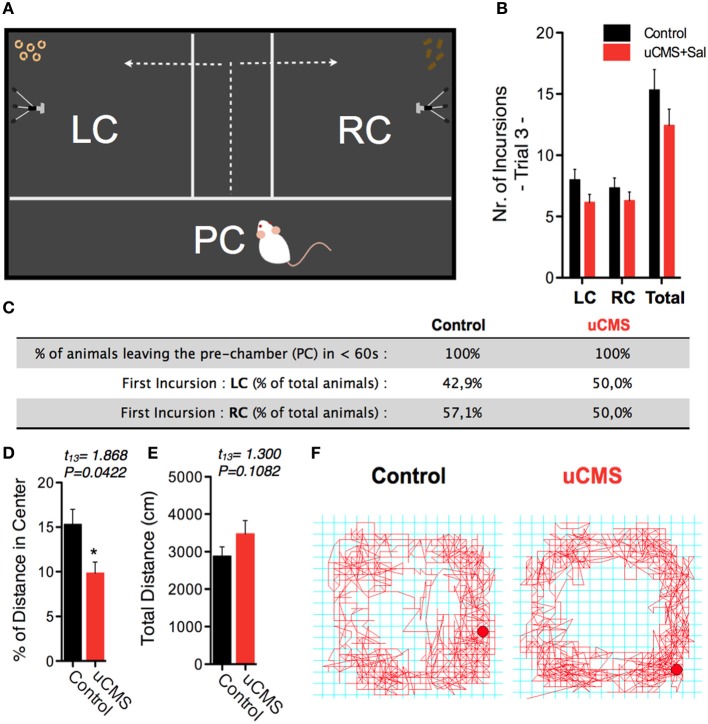
**Exploratory parameters in the SDT. (A,B)** The average number of incursions into the left-chamber (LC; containing sweet pellets) and into the right-chamber (RC; containing regular pellets), during SDT was assessed and no significant differences were found. **(C)** Additional exploratory parameters were assessed in order to exclude a possible bias introduced by hyperanxious behavior of uCMS-exposed animals; the similar exploratory behavior of control and uCMS animals supports the SDT arena as a non-aversive test environment. **(D–F)** Evaluation in the open-field test, further indicates that anxious-like behavior **(D)** did not affect total exploratory activity **(E,F)**. ^*^P < 0.05 Error bars, s.e.m., *n* = 7–9.

Importantly, we detected no significant differences between control and uCMS-exposed animals when analyzing different exploratory parameters, namely the total number of incursions to each food chamber (Figures 3A,B), as well as the average time animals took to leave the PC and start exploring the food chambers, and the choice of the first food chamber to explore (Figure 3C). Analysis of the percentage of distance spent in the center of the arena in the OF test endorsed the hyperanxious-like phenotype of uCMS-exposed animals, that was also found in NSF (Figures 3D–F). Together with the exploratory parameters, this data suggested an effective subtraction of any eventual anxiogenic and neophobic components of the SDT environment.

**Figure 4:**
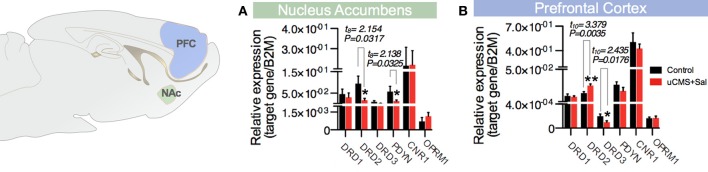
**Expression of molecular mediators of hedonia in the (A) nucleus accumbens (NAc) and (B) prefontal cortex (PFC) by RT-PCR: Dopamine Receptors D1, D2, and D3 (Drd1, Drd2, and Drd3), prodynorphin (Pdyn), Cannabinoid Receptor 1 (Cnr1) and Opioid Receptor μ 1 (Oprm1)**. ^*^P < 0.05, ^**^P < 0.01. Error bars, s.e.m. *t*-test statistical measures for the analysis of uCMS vs. Control is shown on the top of the figures.

Furthermore, we analyzed the expression of molecules described to participate in the mediation of hedonic behavior in the prefrontal cortex (PFC) and in the nucleus accumbens (Nac) (Der-Avakian and Markou, 2012). From the different genes analyzed, we found that the expression of dopamine receptor D2 (Drd2) and prodynorphin (Pdyn) is decreased in the Nucleus Accumbens (Nac) of animals exposed to uCMS (Figure 4A). In the PFC, however, we found Drd2 mRNA levels to be increased in stressed animals, whereas uCMS exposure induced a decrease in dopamine receptor D3 (Drd3) levels in the same region (Figure 4B).

**Figure 5:**
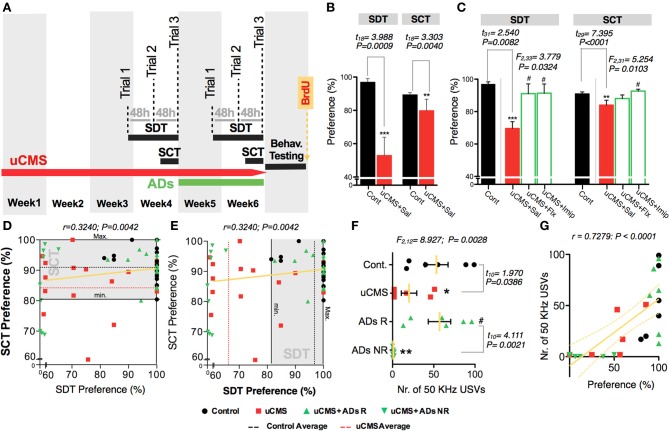
**Measuring the effects of uCMS and antidepressant treatment on anhedonic behavior with the Sweet Drive Test (SDT) and the Sucrose Consumption Test (SCT). (A–C)** Anhedonic behavior was measured before **(B)** and after **(C)** 2 weeks of antidepressant treatment, in the SDT (data from the 3rd trial presented) and in the SCT (*n* = 10–18). **(D,E)** Performances in both tests were correlated (*n* = 68) and the ability of the tests to discriminate anhedonic behavior in untreated uCMS animals is showed as the density of red dots outside the performance range of control animals in each test (depicted as a shaded gray area). **(F)** Total number of 50 KHz vocalizations in control animals (Cont.), uCMS untreated (uCMS) animals and in antidepressant treatment responders (ADs R) and non-responders (ADs NR) (*n* = 6). **(G)** Correlation between the total number of 50 KHz vocalizations and preference values in the 3rd SDT trial (*n* = 24). ^*^P < 0.05, ^**^P < 0.01, ^***^P < 0.001. Error bars, s.e.m. ANOVA statistical measures for the analysis of ADs effect is shown on the top of the figures. ^*^Denotes the effect of uCMS exposure. ^#^Denotes antidepressant effect.

**Figure 6:**
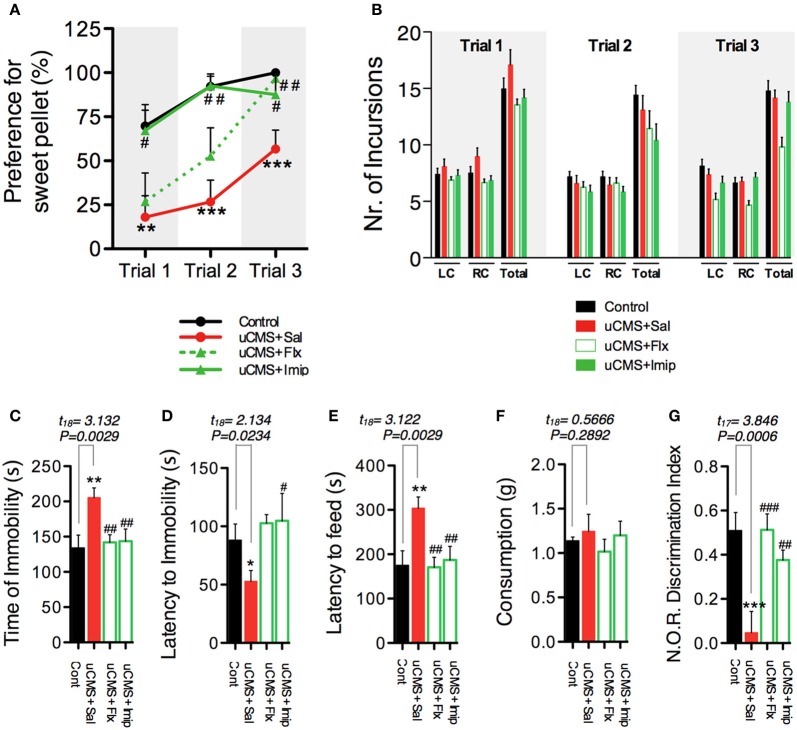
**Additional behavioral parameters determined after 6 weeks of uCMS exposure and ADs treatment. (A)** uCMS exposure induced a significant decrease in preference for sweet pellets along the trials [Trial 1: *t*_(18)_ = 3.011, *P* = 0.0038; Trial 2: *t*_(18)_ = 4.795, *P* < 0.0001; Trial 3: *t*_(18)_ = 4.007, *P* = 0.0004]. This anhedonic profile was effectively reversed by imipramine in the three trials, while the improving effects of fluoxetine were mainly observed in trial 3 [Trial 1: *F*_(2, 27)_ = 3.784, *P* = 0.0356, *Post-hoc*: P_uCMS vs. uCMS + Flx_ > 0.05, P_uCMS vs. uCMS + Imip_ < 0.05; Trial 2: *F*_(2, 27)_ = 7.176, *P* = 0.0032, *Post-hoc*: P_uCMS vs. uCMS + Flx_ > 0.05, P_uCMS vs. uCMS + Imip_ < 0.01; Trial 3: *F*_(2, 27)_ = 5.427, *P* = 0.0104, *Post-hoc*: P_uCMS vs. uCMS + Flx_ < 0.01, P_uCMS vs. uCMS + Imip_ < 0.05]. **(B)** The average number of incursions into the left-chamber (LC; containing sweet pellets) and into the right-chamber (RC; containing regular pellets), during SDT was assessed and no significant differences were found. **(C–G)** Emotional and cognitive domains normally affected in depression were assessed in the Forced Swimming Test **(C,D)** [ANOVA_ADs effect on Time of Immobility:_ *F*_(2, 27)_ = 4.490, *P* = 0.0050, *Post-hoc*: P_uCMS vs. uCMS + Flx_ < 0.01, P_uCMS vs. uCMS + Imip_ < 0.01; ANOVA_ADs effect on Latency to Immobility:_ *F*_(2, 27)_ = 3.710, *P* = 0.0377, *Post-hoc*: P_uCMS vs. uCMS + Flx_ > 0.05, P_uCMS vs. uCMS + Imip_ < 0.05], Novelty Suppressed Feeding **(E,F)** [ANOVA_ADs effect on Latency to Feed:_ *F*_(2, 27)_ = 7.648, *P* = 0.0023, *Post-hoc*: P_uCMS vs. uCMS + Flx_ < 0.01, P_uCMS vs. uCMS + Imip_ < 0.01; ANOVA_ADs effect on Appetite Drive_: *F*_(2, 27)_ = 0.5522, *P* = 0.5823, *Post-hoc*: P_uCMS vs. uCMS + Flx_ > 0.05, P_uCMS vs. uCMS + Imip_ > 0.05] and Novel Object Recognition **(G)** ANOVA_ADs effect on Exploratory Index:_ *F*_(2, 26)_ = 12.45, *P* = 0.0002, *Post-hoc*: P_uCMS vs. uCMS + Flx_ < 0.001, P_uCMS vs. uCMS + Imip_ < 0.01 test paradigms. ^*^P < 0.05, ^**^P < 0.01, ^***^P < 0.001. Error bars, s.e.m. ^*^Denotes the effect of uCMS exposure. ^#^Denotes antidepressant effect. Error bars, s.e.m., *n* = 9–10.

To further evaluate the capacity of SDT to assess anhedonic behavior, in a second approach we tested its sensitivity to detect the reestablishment of normal hedonic behavior, promoted by chronic treatment with ADs from two different classes, fluoxetine (a selective serotonin reuptake inhibitor—SSRI) and imipramine (a tricyclic agent). Moreover, we compared performance in the SDT with the performance of the same animals in the gold-standard test, SCT (Figure 5A). We found that both ADs were able to revert anhedonic behavior measured in the SCT and in the SDT (Figures 5B,C). Although group statistics allowed to discriminate the anhedonic phenotype of the stressed-untreated animal group in both tests, individual score analysis demonstrates that SDT presents a high individual discrimination accuracy (Figure 5D); in fact, most uCMS exposed animals were effectively discriminated based on its preference scores in SDT, with preference values outside the control group score-range (Figure 5E). Furthermore, simultaneous recording of 50 KHz vocalizations allowed to discriminate between uCMS animals that responded to ADs treatment (ADs R) and animals that maintained the anhedonic profile and did not respond to treatment (ADs NR) (Figure 5F). Values of total number of 50 KHz vocalizations correlated positively with performance on SDT (Figure 5G). In complementary behavioral analysis, data shows that treatment with imipramine has a fast action in reverting the pro-anhedonic effects induced by stress exposure, whereas fluoxetine effects are only significant later on, namely on the third trial (Figure 6A). Similarly to what was found in the previous experimental set, no differences were found in the total number of incursions that could potentially undermine SDT interpretation (Figure 6B). Moreover, both ADs effectively reverted the behavioral deficits induced by stress (Figures 6C–G).

**Figure 7:**
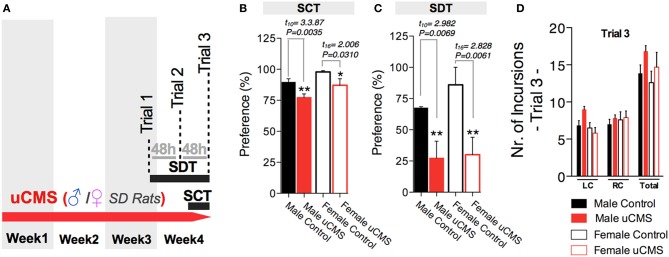
**Measuring anhedonic behavior with the Sweet Drive Test (SDT) and the Sucrose Consumption Test (SCT) in Sprague-Dawley (SD) Rats. (A)** Using the same experimental approach previously used with Wistar-Han rats, male and female SD rats were tested in the SCT and the SDT. **(B)** uCMS exposure induced anhedonic behavior in both male and female animals in the SCT. **(C)** The effects of uCMS on anhedonic behavior were also detected using the SDT, evidencing a marked reduction on preference in both male and female rats. **(D)** No differences on exploratory behavior were observed between groups, during the SDT. ^*^P < 0.05, ^**^P < 0.01. Error bars, s.e.m., *n* = 6–9.

Finally, we tested whether SDT could effectively evaluate anhedonic behavior in a different rat strain—Sprague-Dawley—both in female and male animals (Figure 7A). Using the same protocol of previous experiments, both female and male rats exposed to stress revealed decreased preference values in the SCT and in the SDT (Figures 7B,C), while the exploratory parameters in SDT were similar for all groups (Figure 7D).

## Discussion

Understanding the pathophysiology of anhedonic behavior is currently particularly relevant in different areas of neurosciences research, as it encompasses the symptomatology of different diseases with growing prevalence in modern societies. Studies aiming to characterize this pathological trait in animal models require methods with at least three fundamental characteristics, namely (i) the capacity to provide a solid measure of anhedonic behavior, with high sensitivity to both anhedonic and pro-hedonic stimuli and/or treatments, (ii) the ability to reduce or eliminate the interference of confounding factors that can undermine the interpretation of the obtained results, and (iii) the reliability of the method in different experimental contexts.

A primary concern when designing SDT was to eliminate the interference of common potential confounding factors found in animal testing. Since test performance and exploratory behavior of animals can be largely conditioned by heightened anxiety or neophobia-based test paradigms, it was our aim to subtract these factors as much as possible in the SDT. The use of perforated and transparent chamber dividers was aimed to allow the animals to acquire complete spatial and odor maps of the explorable area in any position, and to reduce neophobic behavior. This apparatus configuration, combined with the absence of white-light illumination and the sound-isolation provided by the transparent lid enclosure was intended to subtract any aversive nature of the testing context, during animal testing. Indeed, despite presenting heightened anxious-like behavior, evidenced in the NSF and OF tests, chronically stressed animals presented an exploratory activity in the SDT that was indistinguishable from control animals, suggesting this test to be uninfluenced by this behavioral dimension. In addition, tests such as the SCT are usually conducted after long periods of simultaneous water and food deprivation (ranging from 18 to 24 h). Starvation may significantly interfere with the test outcome as animals' consumption may not reflect a “hedonistic” choice based on palatability, but rather the necessity of satiety, irrespectively of food palatability or taste. In this study, water was never removed prior testing and animals were allowed to feed freely in their active-nocturnal period (and during the first 2 h of the inactive-diurnal period). In light of the fact that in SDT trials animals had to choose between a sugared-pellet and a pellet made of the same regular food that animals were given in a daily basis, we opted to remove food from animals' cages during the remaining 10 h of the inactive period to preclude pre-conditioning to food odor or taste prior testing.

To validate the SDT, we used an uCMS-based animal model of depression which has been already pre-validated by our lab (Bessa et al., 2009; Mateus-Pinheiro et al., 2013). Animals submitted to uCMS presented several physiological and behavioral deficits encompassed in the spectrum of depressive disorders. Moreover, analysis of the expression of molecules described to participate in the mediation of hedonic behavior revealed a decrease expression of *Drd2* and *Pdyn* in the Nac. Both alterations have already been described in animals presenting increased anhedonic behavior (Blednov et al., 2006; Kruzich et al., 2006) and reinforce the idea that uCMS animals present alterations in the brain systems involved in the regulation of hedonia. Although, to the best of our knowledge, *Drd3* has not been associated with the emergence of anhedonic behavior in pre-clinical studies, we have detected a decrease of *Drd3* in the PFC; whether this finding has a causal relation with the anhedonic phenotype of stressed animals or whether it rather represents an epiphenomenon remains to be elucidated.

Using this animal model of depression, we show that SDT can effectively discriminate anhedonic behavior in stressed animals, based on preference values for sugared-pellets. In addition, we complemented food preference analysis with USVs recording. In fact, USVs have been proved to be a powerful tool to characterize rodents' behavior (Burgdorf et al., 2000, 2007). In particular, recording of 50 KHz frequency vocalizations has been shown to be associated with pleasurable and/or rewarding activities (Borges et al., 2013). Interestingly, we found that the total number of 50 KHz vocalizations emitted by uCMS exposed animals during the test is significantly lower comparatively with control animals. Moreover, the fact that the number of 50 KHz vocalizations correlated positively with preference for sugared pellets indicates that this parameter has a strong potential to complement methods currently available to characterize anhedonic behavior. The combination of these two parameters in the SDT, namely food preference and USVs quantification, offers this test the ability to provide a robust multi-parametric measure of hedonic behavior. Moreover, SDT presented high sensitivity to detect the pro-hedonic effects promoted by two different antidepressants, fluoxetine and imipramine. In fact, when comparing SDT with the *gold-standard* SCT using group statistics, results show that both tests can effectively discriminate the anhedonic behavior induced by stress exposure and the improving effects promoted by ADs. However, an individual analysis of animals' performance shows that SDT has a higher ability to discriminate uCMS-exposed animals, which presented lower preference values, prevalently outside the control group score-range. In addition, data from the second experimental set shows that USVs quantification can also be a valuable tool for the discrimination of animals that responded to treatment from non-responder animals. Once more, the quantification of 50 KHz USVs presented a positive correlation with food preference values. It is also important to mention that the longitudinal analysis of the three SDT trials, revealed that in this particular context of an uCMS-based protocol, anhedonic behavior can be detected as early as in the first trial. Although this observation validates the use of a single-trial protocol of the SDT, the longitudinal analysis of the three trials along the administration of ADs shows that different drugs differ in the time needed to elicit their improving effects, thus justifying the use of a multi-trial approach to accurately report their therapeutic effects. Therefore, a careful decision must be made regarding the use of single- or multi-trial protocol, which must be based on the main purpose and the experimental design of the study to be performed.

Finally, our results showed that SDT could effectively discriminate anhedonic behavior in two rat strains (Wistar-Han and Sprague-Dawley), as well as in male and female genders. Although these results are first indication that this test can be successfully used in different rat strains, it will be also imperative to assess its efficacy in mice models, which are currently widely used to address questions on this topic.

Overall, the present work indicates that the multi-parametric approach of SDT represents a valuable refinement of current methods to assess hedonic behavior in rodents, and can be a robust complement to the characterization of this behavioral dimension, with the potential to be implemented across labs. Improvements such as this, demonstrate that conventional paradigms are flexible to further modification, and may contribute to enhance the utility of animal models of complex neuropsychiatric disorders and to expand current knowledge on the neurobiology of mental disease.

### Conflict of interest statement

The authors declare that the research was conducted in the absence of any commercial or financial relationships that could be construed as a potential conflict of interest.

## Supplementary material

The Supplementary Material for this article can be found online at: at: http://www.frontiersin.org/journal/10.3389/fnbeh.2014.00074/abstract
